# MiR-596 activated by EP300 controls the tumorigenesis in epithelial ovarian cancer by declining BRD4 and KPNA4

**DOI:** 10.1186/s12935-020-01497-0

**Published:** 2020-09-11

**Authors:** Deying Wang, Yulan Cui, Aili Xu, Lin Zhao, Peiling Li

**Affiliations:** Department of Obstetrics and Gynecology, The Second Affiliated Hospital of Harbin Medical University, No. 246, Xuefu Road, Nangang District, Harbin, China

**Keywords:** Epithelial ovarian cancer (EOC), EP300, miR-596, BRD4, KPNA4

## Abstract

**Background:**

Epithelial ovarian cancer (EOC), a subclass of ovarian cancer (OC), is usually diagnosed at advanced stages due to the lack of effective screening means. Mounting reports have disclosed the vitally important roles of microRNAs (miRNAs) in carcinogenesis. Here, we aimed to find out possible miRNAs participating in EOC development.

**Methods:**

qRT-PCR ad western blot respectively examined the mRNA and protein levels of studied genes. CCK-8, colony formation, flow cytometry, TUNEL and spheroid formation assays were appropriately employed for examining cell proliferation, cell cycle, apoptosis and stemness. The interaction between molecules was affirmed by luciferase reporter, RNA pull down and ChIP assays.

**Results:**

In consistent with the observation of a past study, miR-596 expression was relatively low in EOC cells. Up-regulating miR-596 suppressed EOC cell proliferation and stemness. EP300 transcriptionally activated miR-596 to serve as a tumor-repressor in EOC. Then BRD4 and KPNA4, whose knockdown led to restraining effects on cell growth and stemness, were both revealed to be targeted by miR-596 in EOC. Lastly, rescue assays affirmed the tumor-restraining role of miR-596-BRD4/KPNA4 axis in EOC.

**Conclusion:**

EP300-activated miR-596 hampered cell growth and stemness via targeting BRD4 and KPNA4 in EOC, proofing miR-596 as a promising therapeutic target in treating EOC patients.

## Background

Ovarian cancer (OC) is a deadly carcinoma in gynecological system [1, 2], among which epithelial ovarian cancer (EOC) is a typical subclass with the most mortality [3]. Owing to the lack of early symptoms and valid biomarkers, EOC patients often develop into advanced stages when diagnosed, which finally results in disappointing prognosis [4]. Therefore, the identification of available biomarkers for treating EOC patients is the urgent task.

Unlike long noncoding RNAs (lncRNAs), microRNAs (miRs) are noncoding RNAs (ncRNAs) with approximately 20 nt in length [5, 6]. They are widely known to recognize specific sequences in the 3′UTR of target message RNAs (mRNAs) to induce the silence of these mRNAs, so as to elicit restraining or facilitating roles in carcinomas [7]. Moreover, miRNAs with abnormal expression have been proved to affect cellular activities in diverse human cancers [8, 9]. For examples, circRNA AGFG1 acts as a sponge of miR-195-5p to promote triple-negative breast cancer progression through regulating CCNE1 expression [10]; miR-760 suppresses human colorectal cancer cell growth by targeting BATF3AP-1/cyclinD1 signaling [11]; MiR-4319 suppresses the malignancy of triple-negative breast cancer by regulating self-renewal of cancer stem cells [12]. Recently, several researches have demonstrated the tumor suppressive role of miR-596 in malignancies such as gastric cancer [13] and melanoma [14]. Besides, a former report revealed that miR-596 is expressed at a low level in EOC [15]. Yet, no in-depth investigations have been made on the function of miR-596 in EOC.

Our paper aimed to further expose the role and related mechanism of miR-596 in EOC. We firstly affirmed the low expression of miR-596 in EOC cells. Functional assays displayed that overexpression of miR-596 repressed EOC cell proliferation and stemness. To seek the reason for miR-596 down-regulation, we discovered the transcription activation of EP300 on miR-596, which was weakened along with reduced EP300 level in EOC cells. Subsequently, the negative regulation of miR-596 on BRD4 and KPNA4 was revealed. Rescue experiments validated that miR-596 hindered carcinogenesis in EOC through targeting BRD4 and KPNA4.

## Methods

### Cell lines

All cell lines including human EOC cell lines (SKOV3, OVCAR3 and A2780) and human ovarian surface epithelial cell line (HOSEpiC) were procured from Chinese Academy of Sciences (Shanghai, China) and allowed to grow in RPMI-1640 medium. 10% fetal bovine serum (FBS; Gibco, Grand Island, NY) was incorporated into the culture medium for cell logarithmic growth at 37 °C in the air with 5% CO_2_.

### qRT-PCR

Different RNA expression levels were interrogated by qRT-PCR, with GAPDH/U6 as the normalized control using 2^−ΔΔCt^ method. The total RNA sample was collected from A2780 and OVCAR3 cells using Trizol reagent (Invitrogen, Carlsbad, CA) for achieving the first-strand cDNA synthesis using PrimeScript Reverse Transcriptase kit (Takara, Shiga, China). qPCR was implemented with the 5 μL of SYBR Green Master Mix (Invitrogen) on ABI Step One Plus instrument (Applied Biosystems, Foster City, CA). The sequences of primers used were shown in Additional file 1: Table S1.

### Transfection

Confluent EOC cells (A2780 and OVCAR3) were prepared for 48 h of transfection in 6-well culture plates with the help of Lipofectamine2000 (Invitrogen). To elevate or reduce miR-596 expression, miR-596 mimics or inhibitor, as well as the corresponding negative control (miR-NC), were constructed by GenePharma (Shanghai, China). To overexpress EP300, BRD4 or KPNA4, indicated cDNA sequence was inserted into the pcDNA3.1 vector (Invitrogen) to acquire pcDNA3.1/EP300 (EP300), pcDNA3.1/BRD4 (BRD4) or pcDNA3.1/KPNA4 (KPNA4), and the empty pcDNA3.1 vector was seen as the negative control. In the meantime, short hairpin RNAs (shRNAs) specifically against EP300 (shEP300), BRD4 (shBRD4) or KPNA4 (shKPNA4), as well as corresponding nonspecific shRNA control (sh-NC), were obtained from the Ribobio (Guangzhou, China). The sequences of shRNAs were shown in Additional file 2: Table S2.

### CCK-8 assay

For CCK-8 assay, the 96-well culture plates were plated with the transfected EOC cells (1 × 10^3^ per well), followed by adding 10 μL of CCK8 solution (Dojindo, Tokyo, Japan) at the specific times. After 4 h, the microplate reader was used to probe the absorption at 450 nm.

### Colony formation assay

EOC cells were cultured in 6-well plates for about 2 weeks. After that, cells were then rinsed in phosphate buffer saline (PBS) and fixed in 4% paraformaldehyde for 15 min. After crystal violet staining for 30 min, the number of formed colonies was calculated manually.

### Flow cytometry analyses

The apoptosis rate and cell cycle distribution of EOC cells were analyzed with flow cytometry with BD AccuriC6 (BD Biosciences, San Jose, CA) and FlowJo 7.6 software (BD Biosciences). Before that, cell apoptosis was determined using Annexin VePE/7AAD Apoptosis Kit, while cell cycle distribution was tested by PI staining and CycleTEST™ PLUS DNA reagent kit (BD Biosciences).

### TUNEL analysis

After transfection, EOC cells were subjected to In-Situ Cell Death Detection Kit (Roche, Basel, Switzerland) based on the user guide. Cell nuclei were double-stained with TUNEL and DAPI (Beyotime, Shanghai, China). Images of TUNEL-positive cells were captured by fluorescence microscope (NIKON, Tokyo, Japan).

### 3-Dimensional (3-D) spheroid formation assay

The spheroid formation assay was performed in accordance with a former protocol [16]. Simply put, 3 × 10^4^ cells in Matrigel (BD Biosciences, Bedford, MA, USA) with reduced growth factor were added into culture medium with enriched growth factor for two-week incubation, with the medium changed every 2 days. The images of formed spheroids were obtained under a microscope.

### Animal study

Total of 12 nude male mice (aged 6 weeks) were purchased from Slac Laboratories (Shanghai, China) for the in vivo experiments approved by the Ethics Committee of the Second Affiliated Hospital of Harbin Medical University. In short, four kinds of A2780 cells, which were respectively transfected with miR-NC, miR-596 mimics, miR-596 mimics + BRD4 or miR-596 mimics + KPNA4, were inoculated on the backs of the mice. Tumor volume was recorded every 4 days, and mice were sacrificed 4 weeks later. Afterwards, the xenografts were separated from mice, pictured and weighed for further analysis.

### Dual-luciferase reporter assays

For miR-596 promoter analysis, the EOC cells (A2780, OVCAR3) were collected for co-transfection in 24-well plates with reporter vector containing miR-596 promoter, pRL-TK-Renilla and transfection plasmids (shEP300, sh-NC, EP300, pcDNA3.1). The 3′-UTR of BRD4 or KPNA4 sequences with wild-type (WT) or mutant (Mut) miR-596 interacting sites were utilized for inserting to pmirGLO-luciferase vectors (Promega, Madison, WI) to form the reporter vectors BRD4 (WT/Mut) or KPNA4 (WT/Mut), followed by co-transfection into EOC cells with miR-596 mimics or miR-NC. The Dual Luciferase Reporter Assay System (Promega) was implemented 48 h post-transfection to test the luciferase activity.

### ChIP assay

EOC cells treated with 4% formaldehyde were cultured for 10 min to acquire DNA–protein cross-links, which were then sheared into chromatin fragments of 200–500 bp. Immunoprecipitation was performed using the H3K27ac-specific antibody (Millipore, Bedford, MA), with anti-IgG as the negative control. DNA fragments in the final precipitates recovered by magnetic beads were dissected by qRT-PCR.

### RNA pull-down experiments

RNA pull-down assay was implemented by applying the in vitro biotin-labeled RNAs (including sense and antisense miR-596 sequences and a nonsense RNA sequence), termed as Bio-miR-596 sense, Bio-miR-596 antisense (AS) and Bio-NC. After that, the biotinylated RNAs were mixed with cellular extracts and streptavidin-crossed beads, and qRT-PCR analysis of the captured RNAs was followed.

### Western blot

RIPA lysis buffer incorporating protease inhibitor was applied for total protein extraction from A2780 and OVCAR3 cells. Protein samples were then electrophoresed for separation on 10% SDS-PAGE, followed by transferring to polyvinylidene fluoride (PVDF) membranes. After sealing with 5% defatted milk, membranes were blotted with primary antibodies (1:2000; Abcam, Cambridge, MA) against Bax, Bcl2, cleaved-PARP, EP300, BRD4, KPNA4 and GAPDH, and then probed with corresponding secondary antibodies (1:5000; Abcam). The antigen–antibody complex was measured by chemiluminescent detection system (Bio-Rad, Hercules, CA).

### Statistical analyses

Results were given as mean ± standard deviation from 3 various replications. Prism for windows, version 6.0 (GraphPad, San Diego, CA) was implemented for data integration and analysis by use of student’s t test and analysis of variance (ANOVA). The statistical data were thought as significant when P value was less than 0.05.

## Results

### Overexpression of miR-596 hampered the proliferation ability of EOC cells

**Fig. 1:**
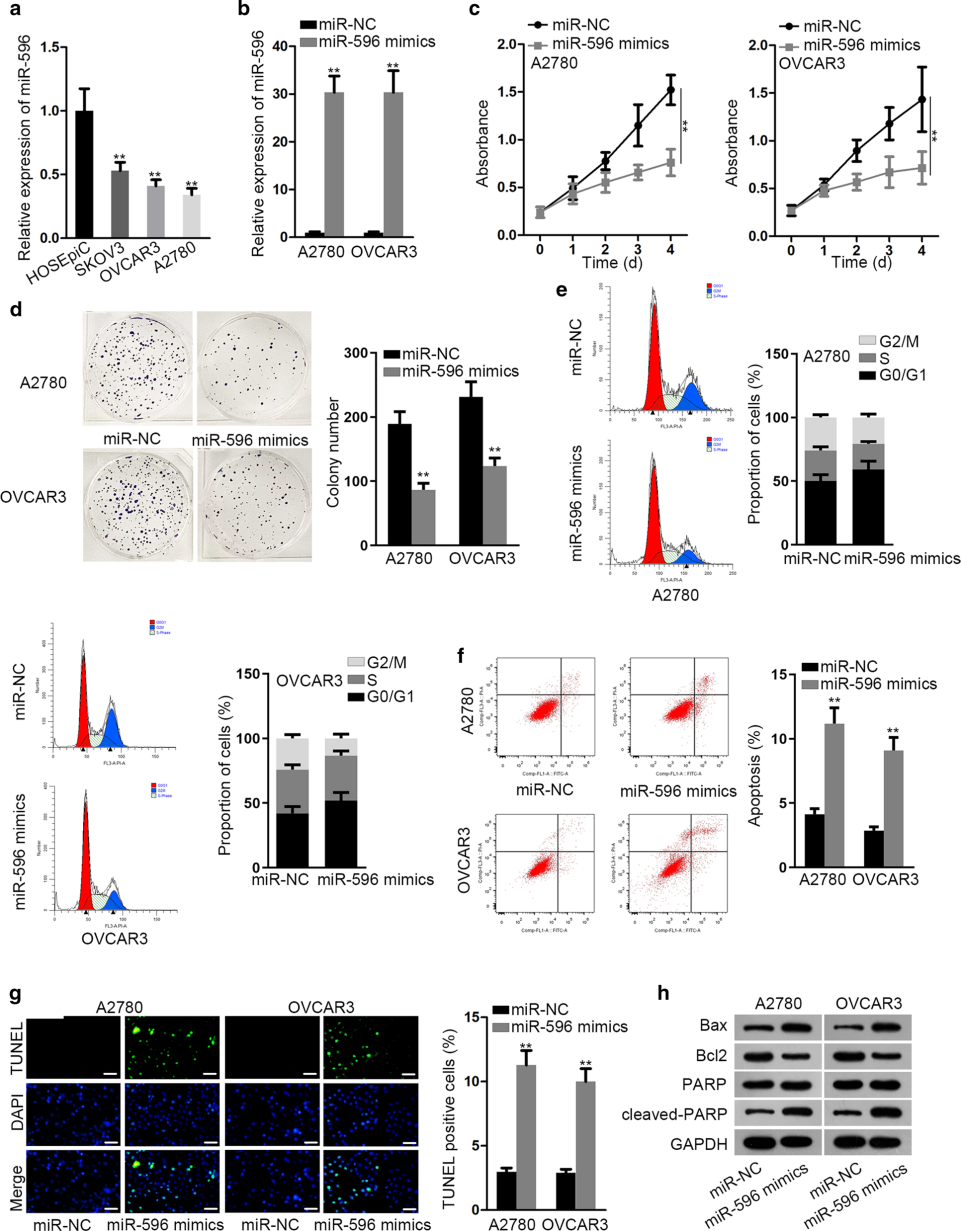
Up-regulation of miR-596 suppressed the proliferation of EOC cells by arresting cell cycle and stimulating cell apoptosis. **a** The enhanced expression of miR-596 in EOC cell lines (SKOV3, OVCAR3, A2780) compared to human ovarian surface epithelial cell line (HOSEpiC) was validated by qRT-PCR. **b** qRT-PCR confirmed the overexpression of miR-596 in A2780 and OVCAR3 cells by transfecting with miR-596 mimics. **c**, **d** The hindered proliferation of A2780 and OVCAR3 cells under miR-596 elevation was indicated by CCK-8 and colony formation assays. **e**, **f** Flow cytometry analyses evidenced that cell cycle was arrested at G0/G1 phases and apoptosis rate was increased under miR-596 upregulation. **g** TUNEL staining assay detected that the percent of TUNEL-positive cells was augmented in miR-596 mimics group relative to miR-NC group. Scale bar = 50 μm. **h** Western blot analyzed that miR-596 enhancement fortified the level of Bax and cleaved PARP and reduced the level of Bcl2. **P < 0.01

Considering the reduced expression of miR-596 in epithelial ovarian cancer (EOC) [15], we decided to further explore the impacts of miR-596 on the biological behaviors of EOC cells. Firstly, qRT-PCR proved that miR-596 expression was indeed decreased in EOC cells in comparison with the normal HOSEpiC cells (Fig. 1a). Then the expression of miR-596 was elevated in A2780 and OVCAR3 cells by miR-596 mimics for subsequent loss-of-function assays (Fig. 1b). The results of CCK-8 assay exhibited that cell viability was significantly repressed by miR-596 overexpression (Fig. 1c). Meanwhile, cell proliferation was distinctly inhibited when miR-596 was up-regulated (Fig. 1d). As for cell cycle, flow cytometry analysis was performed and the outcomes demonstrated that the proportion of cells at G0/G1 phase was increased in miR-596 mimics groups (Fig. 1e), suggesting the arrest of cell cycle induced by enhanced miR-596. Nonetheless, cell apoptosis was evidently induced by ectopic expression of miR-596, since more apoptotic cells and TUNEL positive cells were observed after the transfection of miR-596 mimics (Fig. 1f, g). Additionally, western blot analyzed that Bax and cleaved-PARP protein levels were improved while Bcl2 protein level was lessened after miR-596 was increased (Fig. 1h). Afterwards, we further tested the impact of miR-596 on EOC cell stemness, which is a specific character of malignant cells [17]. As anticipated, the number of spheroids formed in these two EOC cells was apparently declined under ectopic miR-596 expression (Additional file 3: Figure S1A). Altogether, miR-596 hampered cell growth and stemness in EOC.

### EP300 activated miR-596 expression at transcriptional level

**Fig. 2:**
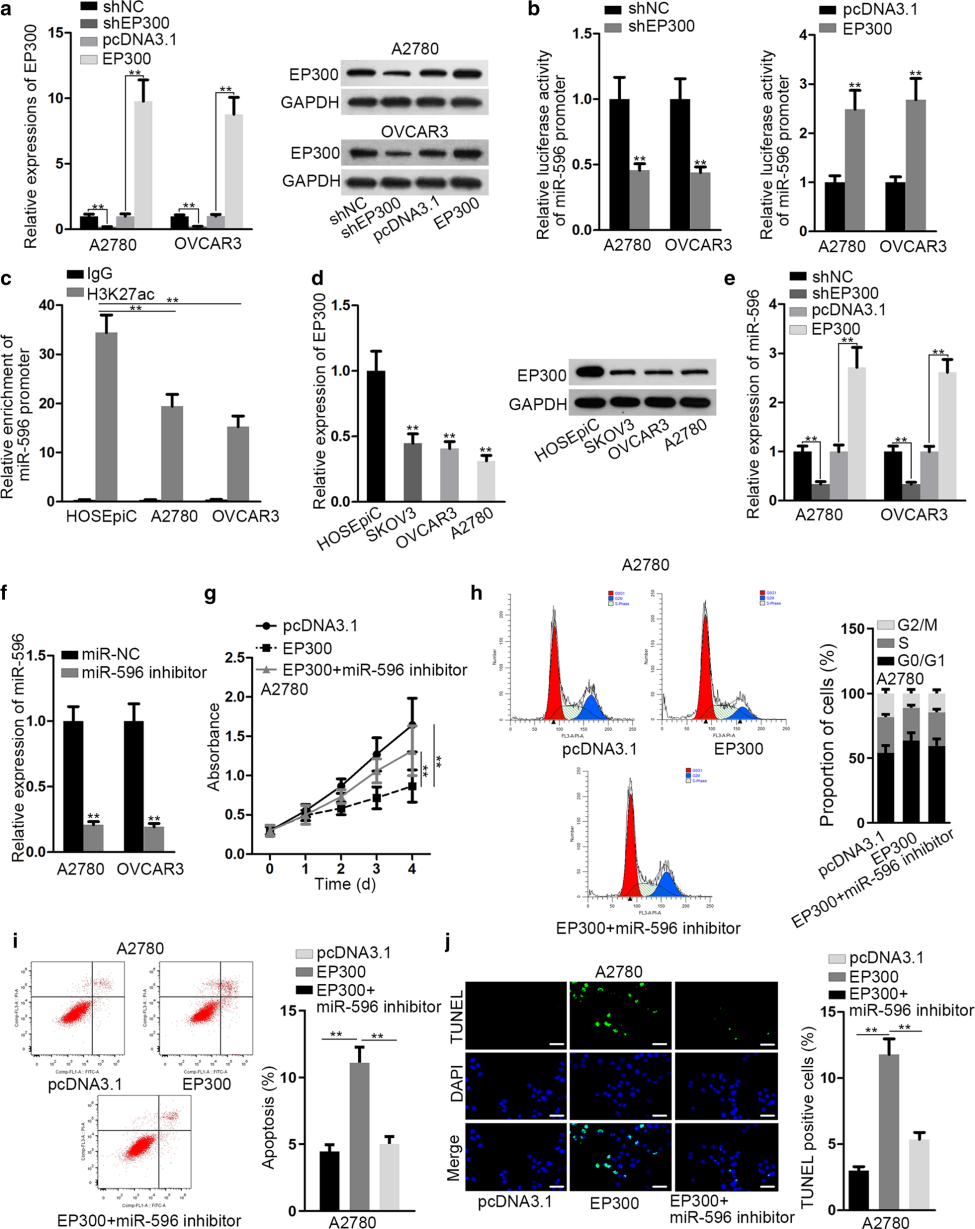
EP300 transcriptionally stimulated miR-596 to restrain the growth of EOC cells. **a** qRT-PCR and western blot verified that EP300 expression in A2780 and OVCAR3 cells was silenced by shEP300 but elevated by pcDNA3.1/EP300 (EP300). **b** Dual-luciferase reporter assay detected that the luciferase activity of miR-596 promoter was decreased or increased after EP300 was inhibited or overexpressed. **c** ChIP assay validated that the high H3K27ac status in miR-596 promoter in EOC cells compared with that in normal HOSEpiC cells. **d** EP300 expression at mRNA and protein levels was reduced in EOC cells relative to HOSEpiC cells, as assessed by qRT-PCR and western blot. **e** qRT-PCR confirmed that miR-596 level was inhibited or enhanced in EOC cells in response to EP300 depletion or overexpression, separately. **f** qRT-PCR proved the successful inhibition of miR-596 in EOC cells by miR-596 inhibitor. **g** Cell viability was controlled by EP300 upregulation but recovered after miR-496 suppression, as monitored by CCK-8 assay. **h**, **i** Flow cytometry analyses indicated that EP300 induced cell cycle arrest and apoptosis, while such effects were offset by inhibited miR-596. **j** TUNEL assay results proved that EP300 augmented the percentage of TUNEL-positive cells, while inhibiting miR-596 could counteract such influence. Scale bar = 50 μm. **P < 0.01

Considering the down-regulation of miR-596 in EOC, we assumed that there might be some transcription factors affecting the transcription activity of miR-596. From UCSC genome browser, we observed that EP300, which could act as a histone acetyltransferase to modulate transcription activation, was a possible transcription factor of miR-596. Hence, we sought to investigate the association between EP300 and miR-596 in EOC. Prior to that, we validated the knockdown and overexpression efficiencies of EP300 expression in A2780 and OVCAR3 cells (Fig. 2a). The results of luciferase reporter assay indicated that the luciferase activity of miR-596 promoter was impaired by silenced EP300 but strengthened by overexpressed EP300 (Fig. 2b). Since EP300 has been indicated to function through modulating H3K27 acetylation (H3K27ac) on gene promoter [18, 19], we then assessed H3K27ac status of miR-596 promoter via ChIP assay. It manifested that less H3K27ac in miR-596 promoter was observed in A2780 and OVCAR3 cells than that in control cells (Fig. 2c). Moreover, EP300 expression at both mRNA and protein levels was overtly underexpressed in EOC cells relative to normal HOSEpiC cells (Fig. 2d). Importantly, miR-596 expression was accordantly lessened or enlarged after EP300 expression was silenced or enhanced (Fig. 2e). To view the impact of EP300/miR-596 axis on EOC cellular activities, we conducted subsequent rescue assays in EP300-overexpressed cells via suppressing miR-596 by miR-596 inhibitor (Fig. 2f). Consequently, we unveiled that the hindered viability of EOC cells with enhanced EP300 was regained when miR-596 was further inhibited (Fig. 2g). Besides, cell cycle progression that was previously arrested by EP300 upregulation was recovered in response to miR-596 inhibition (Fig. 2h). Also, the accelerating effect of EP300 overexpression on cell apoptosis was offset by miR-596 inhibitor (Fig. 2i, j). More importantly, EP300 elevation resulted in hampered spheroid formation ability of both EOC cells, while such inhibitory impact was then offset by miR-596 suppression (Additional file 3: Figure S1B). These data suggested that EP300 promoted miR-596 expression to restrain cell growth in EOC.

### MiR-596 targeted BRD4 and KPNA4 in EOC

**Fig. 3:**
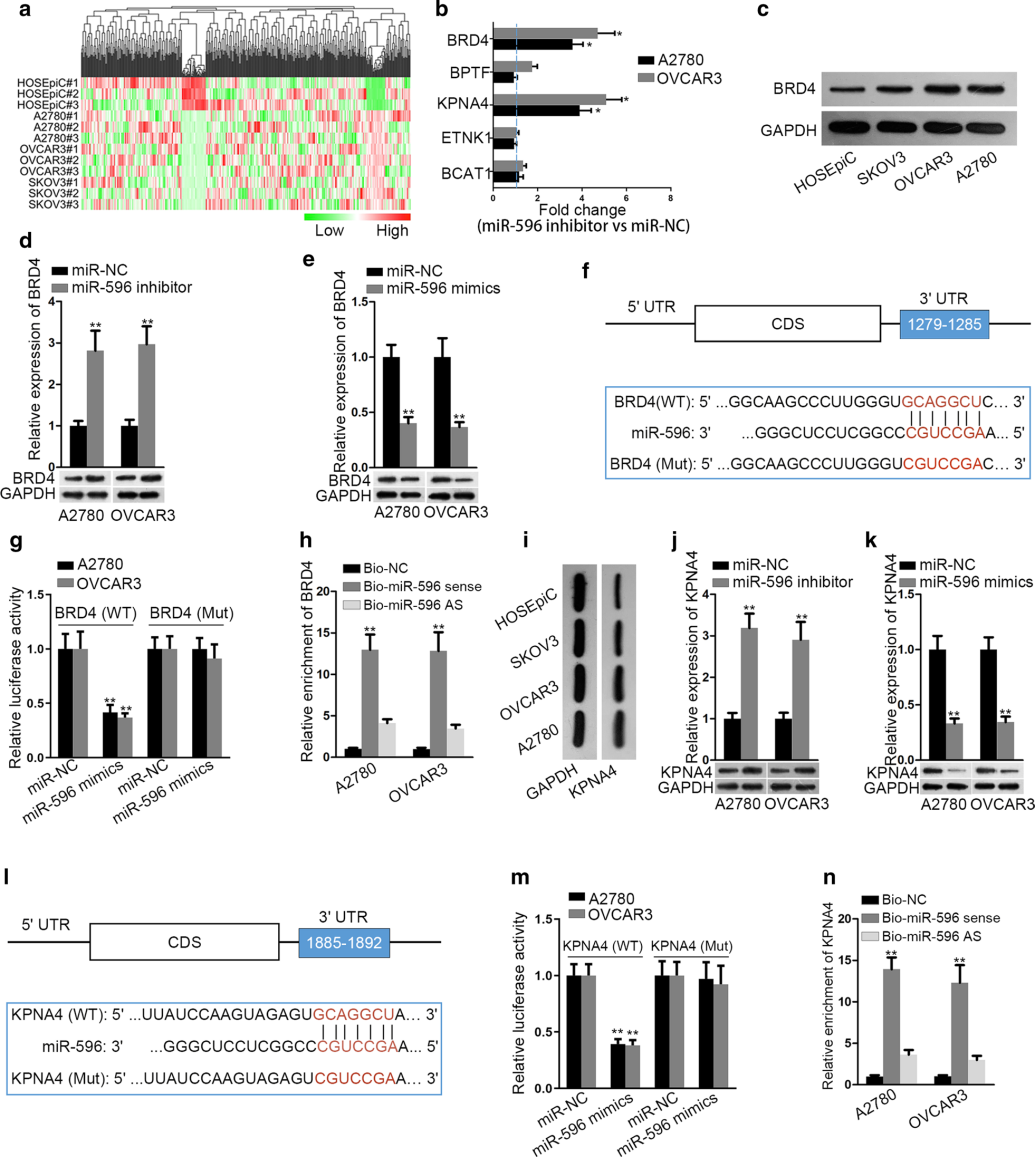
BRD4 and KPNA4 acted as two targets of miR-596 in EOC. **a** As indicated in the heatmap, qRT-PCR analysis unveiled the expression pattern of possible miR-596 targets in EOC cells relative to HOSEpiC cells. **b** The outcomes of qRT-PCR proved that only BRD4 and KPNA4 were targeted by miR-596 in both A2780 and OVCAR3 cells. **c** Heightened protein levels of BRD4 in EOC cells was disclosed by western blot. **d**, **e** The results of qRT-PCR and western blot testified the enhanced or reduced mRNA and protein levels of BRD4 in A2780 and OVCAR3 cells responding to miR-596 inhibitor or miR-596 mimics. **f** The sequences of BRD4 3′UTR with wild-type or mutant miR-596 binding sites were designed for dual-luciferase reporter assays. **g** As examined by luciferase reporter assay, the luciferase activity of BRD4 (WT) was declined whereas that of BRD4 (Mut) was not affected in EOC cells with miR-596 upregulation. **h** The high enrichment of BRD4 in the complex pulled down by biotinylated miR-596 sense was examined by RNA pull-down assay. **i** Elevated protein levels of KPNA4 in EOC cells was validated by western blot. **j**, **k** The outcomes of RT-PCR and western blot uncovered that KPNA4 expression in EOC cells was enhanced by miR-596 inhibitor and decreased by miR-596 mimics. **l** The sequences of KPNA4 3′UTR containing wild-type or mutant miR-596 binding sites were designed for dual-luciferase reporter assays. **m** Luciferase reporter assay results indicated that miR-596 interacted with KPNA4 at the predicted binding sites. **n** The high enrichment of KPNA4 in the complex captured by biotinylated miR-596 sense was validated by RNA pull down assay plus qRT-PCR analysis. *P < 0.05 and **P < 0.01

Thereafter, we screened the targets of miR-596 via mirDIP database, and then analyzed the expression of top 500 targets in three EOC cells relative to normal HOSEpiC cells. As presented in the heatmap, five among them showed high expression in these EOC cells BRD4, BPTF, KPNA4, ETNK1 and BCAT1 (Fig. 3a). However, only two of the five candidates, BRD4 and KPNA4, exhibited elevated expression in both A2780 and OVCAR3 cells under miR-596 inhibition (Fig. 3b), hinting that BRD4 and KPNA4 were the most probable targets of miR-596 in EOC. Further, western blotting elucidated the high expression of BRD4 protein in EOC cells (Fig. 3c). Moreover, inhibition or elevation of miR-596 augmented or declined the mRNA and protein expression of BRD4 in the two EOC cells, respectively (Fig. 3d, e). Next, the binding sites between BRD4 and miR-596, as well as the mutant sequence of BRD4 were represented in Fig. 3f. After that, luciferase reporter and RNA pull-down assays were utilized to confirm the interplay between miR-596 and BRD4. Results indicated that in both A2780 and OVCAR3 cells, miR-596 up-regulation only impaired the luciferase activity of reporters with wild-type BRD4 sequence (Fig. 3g). Also, BRD4 mRNA could merely be pulled down by Bio-miR-596 sense probe (Fig. 3h). As for KPNA4, we observed that KPNA4 protein was markedly elevated in EOC cells (Fig. 3i). Further, KPNA4 expression at mRNA and protein levels displayed similar changes as BRD4 in response to miR-596 inhibition or miR-596 upregulation (Fig. 3j, k). Likewise, the luciferase activity of KPNA4 (WT) was specifically decreased by miR-596 mimics and KPNA4 mRNA was merely pulled down by biotinylated sense miR-596 (Fig. 3l–n). These findings told us that BRD4 and KPNA4 were two targets of miR-596 in EOC.

### Inhibiting BRD4 and KPNA4 hindered EOC cell growth

**Fig. 4:**
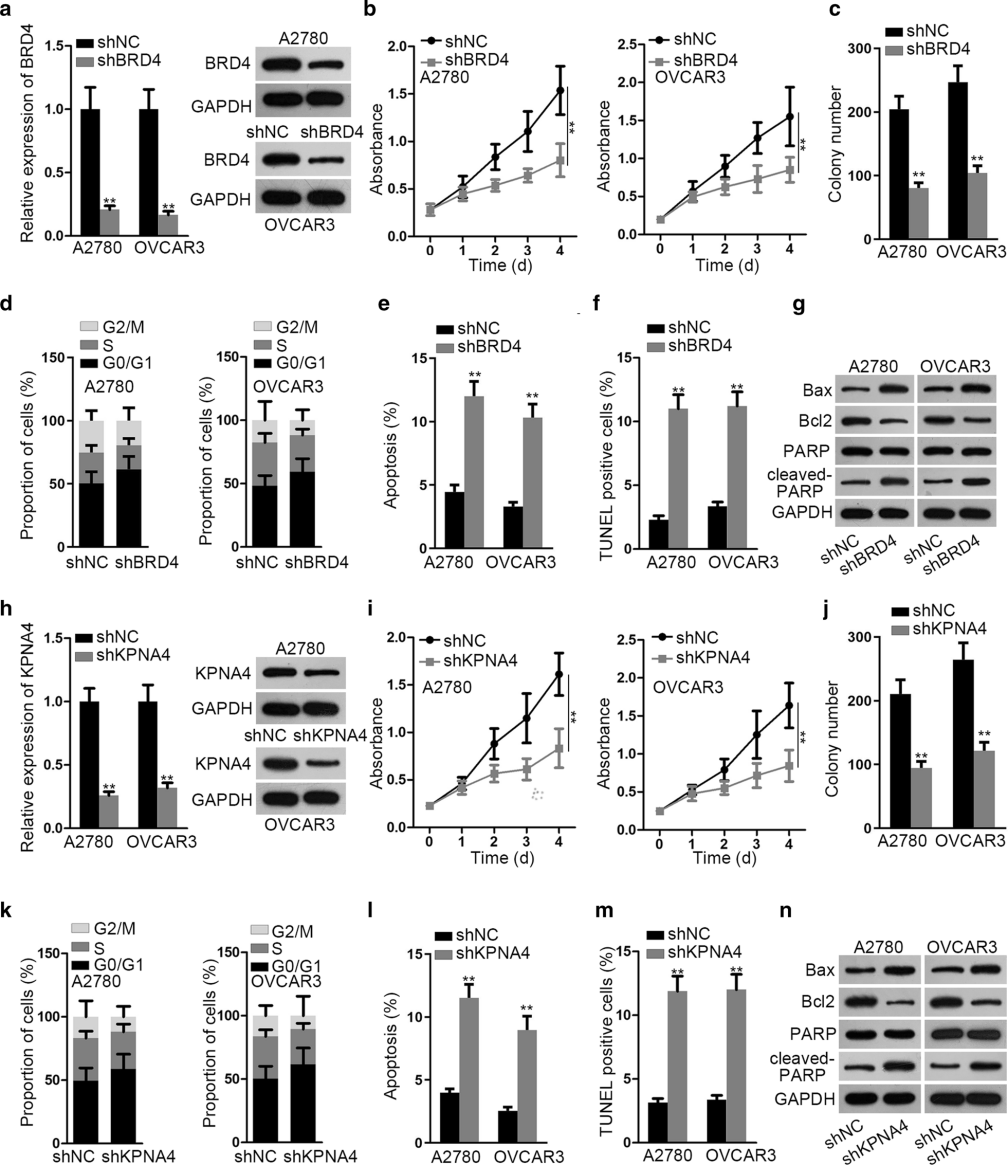
BRD4 or KPNA4 knockdown served restraining effects on the growth of EOC cells. **a** The transfection of shBRD4 suppressed BRD4 expression at mRNA and protein levels in A2780 and OVCAR3 cells, as detected via qRT-PCR and western blot. **b**–**g** The results of functional assays demonstrated that depleted BRD4 impaired EOC cell proliferation, blocked cell cycle progression, and facilitated cell apoptosis. **h** As examined by qRT-PCR and western blot, the expression of KPNA4 was weakened by shKPNA4 compared to that in the control group (shNC). **i**–**n** The suppressive impact of KPNA4 knockdown on the growth of A2780 and OVCAR3 cells was assessed by CCK-8, colony formation and TUNEL assays and flow cytometry and western blot analyses. **P < 0.01

In order to probe the functional role of BRD4 and KPNA4 in EOC, loss-of-function experiments were conducted. BRD4 mRNA and protein expression was firstly downregulated in A2780 and OVCAR3 cells by transfecting with shBRD4 (Fig. 4a). Then, we observed that after BRD4 was silenced, cell viability was weakened and the number of colonies formed by these cells was reduced (Fig. 4b, c), which meant that cell proliferation was controlled under BRD4 deficiency. Besides, the loss of BRD4 led to cell cycle arrested at G0/G1 phase (Fig. 4d). On the contrary, cell apoptosis was activated after BRD4 knockdown, which was affirmed by increased apoptotic rate and TUNEL positive cell proportion (Fig. 4e, f). Moreover, such phenomenon was further proved by the increased Bax and cleaved-PARP protein levels and reduced Bcl2 protein levels in face of BRD4 depletion (Fig. 4g). In the meantime, it manifested that the absence of BRD4 mitigated the stemness of EOC cells (Additional file 3: Figure S1C). Similarly, we also uncovered that KPNA4 silence inhibited cell proliferation, induced cell cycle arrest, accelerated cell apoptosis and impaired cell stemness in EOC (Fig. 4h–n and Additional file 3: Figure S1D). All in all, BRD4 and KPNA4 served restraining roles in EOC cell growth.

### Ectopic expression of BRD4 or KPNA4 reversed the repressive influence of elevated miR-596 on EOC tumorigenesis

**Fig. 5:**
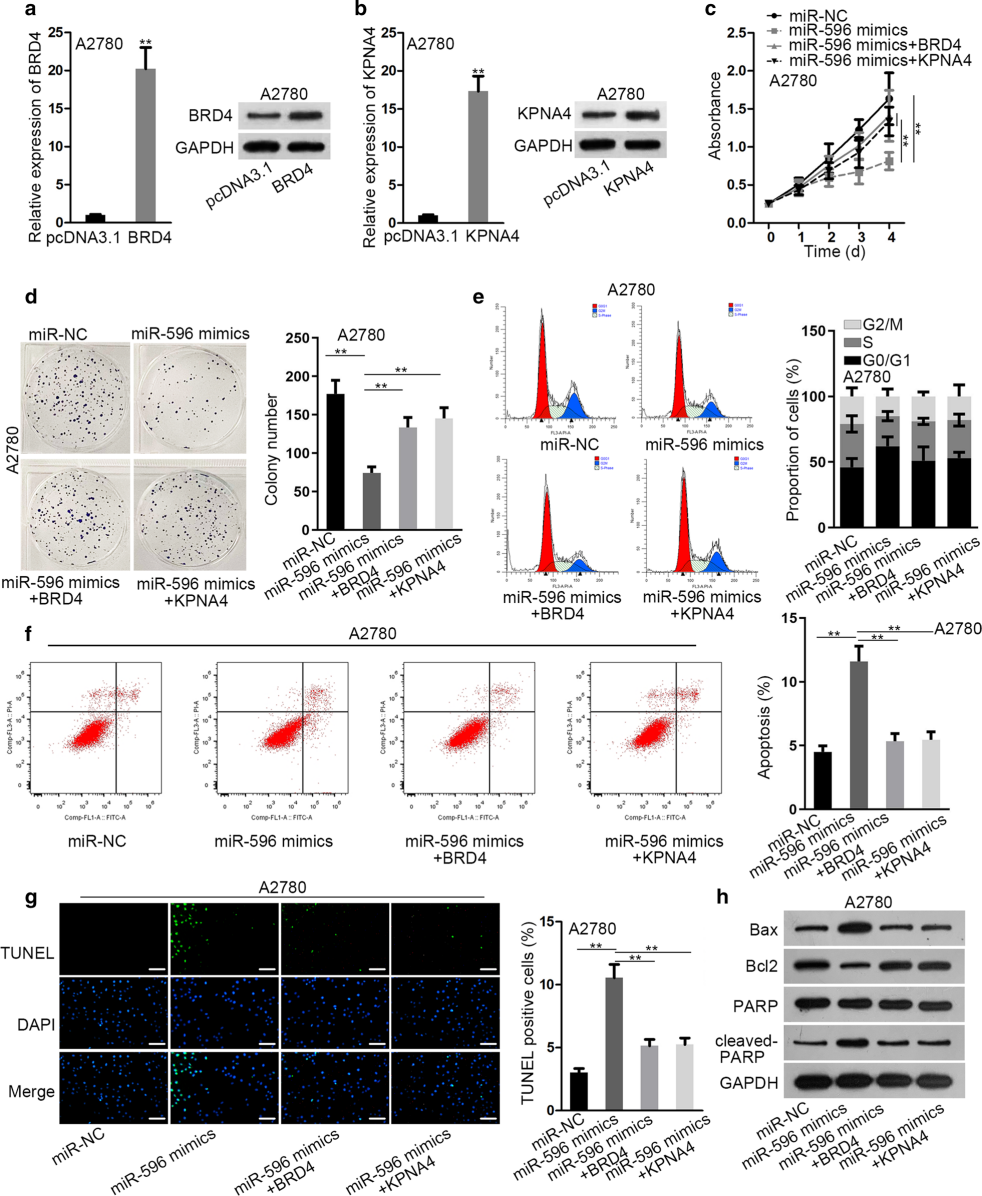
MiR-596 regulated cell proliferation, cell cycle and apoptosis in EOC via targeting BRD4 and KPNA4. **a**, **b** Elevation on the mRNA and protein levels of BRD4 or KPNA4 severally induced by transfection of pcDNA3.1/BRD4 (BRD4) or pcDNA3.1/KPNA4 (KPNA4) in A2780 cells was determined by qRT-PCR and western blot. **c**, **d** The results of CCK-8 and colony formation assays proved that miR-596 upregulation-restrained cell proliferation was reversed by overexpressing BRD4 or KPNA4. **e**–**h** The normalization of BRD4 or KPNA4 overexpression on cell cycle and apoptosis of miR-596-enhanced A2780 cells were confirmed by flow cytometry, TUNEL staining (Scale bar = 50 μm) and western blot. **P < 0.01

Before the rescue experiments, BRD4 and KPNA4 were separately overexpressed in A2780 cells via transfecting with particular pcDNA3.1 overexpressing vectors (Fig. 5a, b). Thereafter, we disclosed that cell viability impaired by miR-596 mimics was rescued by upregulating either BRD4 or KPNA4 (Fig. 5c). Meanwhile, the reduced number of colonies due to miR-596 upregulation was recovered by enhancement of BRD4 or KPNA4 (Fig. 5d). Also, the arrest of cell cycle resulted from miR-596 upregulation was relieved in response to further overexpression of BRD4 or KPNA4 (Fig. 5e). In addition, according to the outcomes of flow cytometry analysis and TUNEL assay, the active impact of miR-596 overexpression on cell apoptosis was neutralized when BRD4 or KPNA4 was augmented (Fig. 5f–g). Meanwhile, it was evidenced that the effects of miR-596 up-regulation on the level of several apoptosis-related proteins (Bax, Bcl2 and cleaved-PARP) were counteracted after increasing the expression of BRD4 or KPNA4 (Fig. 5h). Further, we proved that the suppressed stemness in miR-596-upregulated EOC cells was recovered under the overexpression of BRD4 or KPNA4 (Additional file 3: Figure S1E). To further proof the significance of miR-596/BRD4/KPNA4 axis in EOC tumorigenesis, we then carried out in vivo experiments. As a result, we viewed that the size, growth rate and weight of tumors came from miR-596-elevated cells were all lessened compared to control group, whereas upregulation of BRD4 or KPNA4 could reverse above reductions (Additional file 3: Figure S1F, G). In summary, BRD4 and KPNA4 was implicated in miR-596-controlled tumorigenesis in EOC.

## Discussion

Mounting miRs have been indicated to be dysregulated in EOC to elicit either promotive or repressive effects [20–22]. For example, miR-506 restrains multiple targets associated with epithelial-to-mesenchymal transition (EMT) in EOC [23]. MiR-211 hinders cell proliferation by blocking cell-cycle progression in EOC via Cyclin D1 and CDK6 [24]. In this paper, we focused on the role of miR-596 in EOC, given that miR-596 was reported to be under-expressed in EOC [15]. Consistently, we also proved the down-regulation of miR-596 in EOC cells. More importantly, our work detected that upregulating miR-596 hindered cell proliferation via hampering cell cycle progression and accelerating cell apoptosis, and also impaired cell stemness in EOC. From these findings, we firstly identified miR-596 as a novel tumor-inhibitor in EOC, which was similar to several previous reports regarding miR-596 function in malignancies including melanoma [14], oral cancer [25] and gastric cancer [13].

In order to find the possible upstream molecules regulating miR-596 in EOC, we browsed UCSC genome browser and figured out several transcription factors of miR-596. Herein, we validated the binding between EP300 and miR-596 promoter, and confirmed the positive regulation of EP300 on miR-596 in EOC cells. These results proved E1A-Binding Protein P300 (EP300) as the transcription activator of miR-596 in EOC. Interestingly, EP300, which acts as a histone acetyltransferase to activate gene transcription [26, 27], has been previously reported to exert an anti-cancer function in tumors like breast cancer [28, 29]. However, some literatures also suggested EP300 as a tumor-promoter in several malignancies [30, 31]. In this work, we unveiled that EP300 was downregulated in EOC cells, which was consistent with the discovery of a former study [32]. Moreover, it was elucidated that EP300 upregulation mitigated EOC cell growth and stemness, indicating it served an anti-cancer part in EOC. Further, the inhibitory impact of EP300 on EOC cellular behaviors could be counteracted by miR-596 suppression, highlighting the tumor-repressive role of EP300/miR-596 signaling in EOC.

Next, we attempted to discover the downstream mechanism whereby miR-596 worked in EOC. Fortunately, through bioinformatics analysis and experimental detections, we unveiled bromodomain containing 4 (BRD4) and karyopherin subunit alpha 4 (KPNA4) as the most potential two targets of miR-596 in EOC. Currently, several reports have elucidated the high expression and promotive function of BRD4 and KPNA4 in some diseases. For instance, NSD3-BRD4-CHD8 pathway functions in pelvic high-grade serous carcinomas originated from tubo-ovarian and endometrial [33]. Of note, BRD4 is predicted as a novel therapeutic target for regulating Notch3 signaling in OC [34]. Also, MiR-3619-5p inhibits cell proliferation and cisplatin resistance in cutaneous squamous cell cancer by targeting KPNA4 [35]. Increased miR-181 s reverses EMT via decreasing KPNA4 in glioblastoma [36]. In the present research, we similarly found the oncogenic roles of BRD4 and KPNA4 in EOC, evidenced by that silencing BRD4 or KPNA4 led to abrogated EOC cell growth and stemness. In the end, the rescue assays conducted both in vitro and in vivo verified that miR-596 restrained tumorigenesis in EOC via modulating BRD4/KPNA4 axis.

## Conclusion

These findings implied that EP300-activated miR-596 negatively regulated cell growth and stemness in EOC through declining BRD4 and KPNA4. Although the conclusion provided promising therapeutic targets for treating EOC patients, future efforts are still necessary for deep comprehension of EOC pathology.

## Supplementary information

**Additional file 1: Table S1.** The sequences of primers used in qRT-PCR.

**Additional file 2: Table S2.** The sequences of shRNAs.

**Additional file 3: Figure S1. A.** Spheroid formation assay results disclosed the reduced stemness of A2780 and OVCAR3 cells under miR-596 upregulation. Scale bar = 100μm. **B.** As proved by the results of spheroid formation assay, EP300 elevation hampered EOC cell stemness while inhibiting miR-596 recovered such impairment. Scale bar = 100 μm. **C-D.** Silencing BRD4 or KPNA4 led to abrogated stemness in EOC cells, as assessed by spheroid formation assay. **E.** The outcomes of spheroid formation assay evidenced that overexpression of BRD4 or KPNA4 countervailed the repression of upregulated miR-596 on the stemess of A2780 cells. Scale bar = 100 μm. **F-G.** Results of in vivo experiments unmasked that miR-596 upregulation blocked tumor growth rate and led to lessened tumor size and weight, which were all offset after overexpressing BRD4 or KPNA4. **P < 0.01.

## Data Availability

All data collected have been presented in the manuscript and the associated additional files.

## Abbreviations

EOC: epithelial ovarian cancer
OC: ovarian cancer
ncRNAs: noncoding RNAs
miRs: microRNAs
lncRNAs: long noncoding RNAs
mRNAs: message RNAs
FBS: fetal bovine serum
EP300: pcDNA3.1/EP300
BRD4: pcDNA3.1/BRD4
KPNA4: pcDNA3.1/KPNA4
shRNAs: short hairpin RNAs
PBS: phosphate buffer saline
WT: wild-type
Mut: mutant
AS: antisense
PVDF: polyvinylidene fluoride
ANOVA: analysis of variance
EMT: epithelial-to-mesenchymal transition
BRD4: Bromodomain containing 4
KPNA4: karyopherin subunit alpha 4
