# Supplementary figures and images for: MiR-596 activated by EP300 controls the tumorigenesis in epithelial ovarian cancer by declining BRD4 and KPNA4
Source: Cancer Cell Int. 2020 Sep 11;20:447. doi: 10.1186/s12935-020-01497-0 (PMC7488530; doi:10.1186/s12935-020-01497-0)

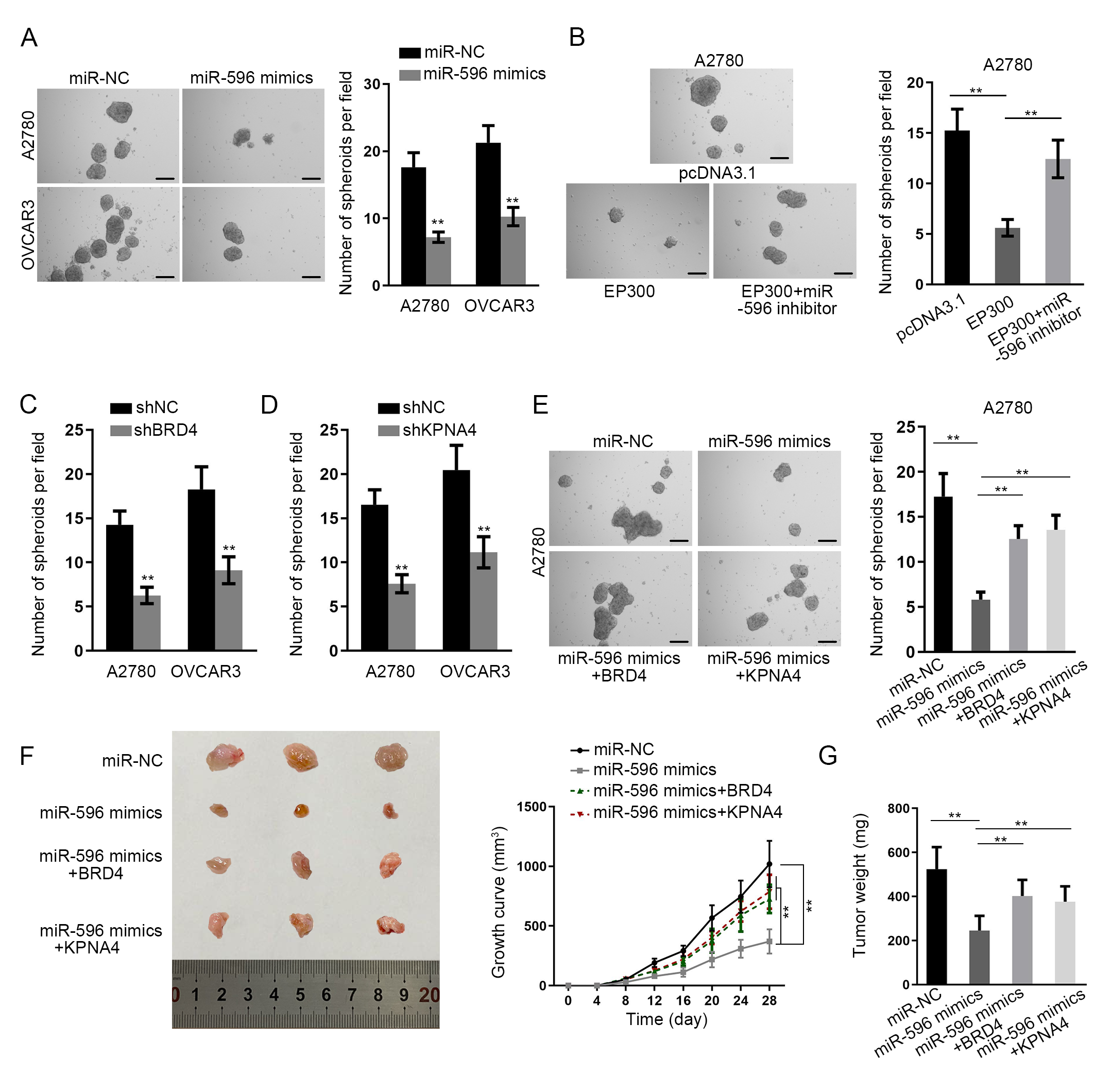

Supplement: Supplementary file 3 — Additional file 3: Figure S1. A. Spheroid formation assay results disclosed the reduced stemness of A2780 and OVCAR3 cells under miR-596 upregulation. Scale bar = 100μm. B. As proved by the results of spheroid formation assay, EP300 elevation hampered EOC cell stemness while inhibiting miR-596 recovered such impairment. Scale bar = 100 μm. C-D. Silencing BRD4 or KPNA4 led to abrogated stemness in EOC cells, as assessed by spheroid formation assay. E. The outcomes of spheroid formation assay evidenced that overexpression of BRD4 or KPNA4 countervailed the repression of upregulated miR-596 on the stemess of A2780 cells. Scale bar = 100 μm. F-G. Results of in vivo experiments unmasked that miR-596 upregulation blocked tumor growth rate and led to lessened tumor size and weight, which were all offset after overexpressing BRD4 or KPNA4. **P < 0.01. [file 12935_2020_1497_MOESM3_ESM.tif]
